# Early arthritis induces disturbances at bone nanostructural level reflected in decreased tissue hardness in an animal model of arthritis

**DOI:** 10.1371/journal.pone.0190920

**Published:** 2018-01-09

**Authors:** Bruno Vidal, Rita Cascão, Mikko A. J. Finnilä, Inês P. Lopes, Simo Saarakkala, Peter Zioupos, Helena Canhão, João E. Fonseca

**Affiliations:** 1 Instituto de Medicina Molecular, Faculdade de Medicina, Universidade de Lisboa, Lisboa, Portugal; 2 Research Unit of Medical Imaging, Physics and Technology, Faculty of Medicine, University of Oulu, Oulu, Finland; 3 Department of Applied Physics, University of Eastern Finland, Kuopio, Finland; 4 Medical Research Center Oulo, Oulu University, Oulu, Finland; 5 Department of Diagnostic Radiology, Oulu University Hospital, Oulu, Finland; 6 Biomechanics Labs, Cranfield Forensic Institute, Cranfield University, Defence Academy of the UK, Shrivenham, United Kingdom; 7 EpiDoC Unit, CEDOC, NOVA Medical School, NOVA University, Lisbon, Portugal; 8 Rheumatology Department, Centro Hospitalar de Lisboa Norte, EPE, Hospital de Santa Maria, Lisbon Academic Medical Centre, Lisbon, Portugal; Indiana University Purdue University at Indianapolis, UNITED STATES

## Abstract

**Introduction:**

Arthritis induces joint erosions and skeletal bone fragility.

**Objectives:**

The main goal of this work was to analyze the early arthritis induced events at bone architecture and mechanical properties at tissue level.

**Methods:**

Eighty-eight Wistar rats were randomly housed in experimental groups, as follows: adjuvant induced arthritis (AIA) (N = 47) and a control healthy group (N = 41). Rats were monitored during 22 days for the inflammatory score, ankle perimeter and body weight and sacrificed at different time points (11 and 22 days post disease induction). Bone samples were collected for histology, micro computed tomography (micro-CT), 3-point bending and nanoindentation. Blood samples were also collected for bone turnover markers and systemic cytokine quantification.

**Results:**

At bone tissue level, measured by nanoindentation, there was a reduction of hardness in the arthritic group, associated with an increase of the ratio of bone concentric to parallel lamellae and of the area of the osteocyte lacuna. In addition, increased bone turnover and changes in the microstructure and mechanical properties were observed in arthritic animals, since the early phase of arthritis, when compared with healthy controls.

**Conclusion:**

We have shown in an AIA rat model that arthritis induces very early changes at bone turnover, structural degradation and mechanical weakness. Bone tissue level is also affected since the early phase of arthritis, characterized by decreased tissue hardness associated with changes in bone lamella organization and osteocyte lacuna surface. These observations highlight the pertinence of immediate control of inflammation in the initial stages of arthritis.

## Introduction

Rheumatoid arthritis (RA) is the most common chronic inflammatory joint disease, affecting about 1% of the world population [1]. RA is characterized by synovial hyperplasia caused by a large proliferative cellular infiltrate of leukocytes and high expression levels of proinflammatory cytokines [2]. As RA progresses there is marked articular destruction and decreased joint mobility with radiological evidence of bone erosion within 2 years of disease onset [3]. In addition, osteoporosis is a common finding in patients with RA [4] and is responsible for increased rates of vertebral and hip fractures in these patients [5,6]. RA is associated with an augmented expression of the receptor activator of RANKL (receptor activator of nuclear factor kappa–B ligand, NF-KB ligand) and low levels of its antagonist, osteoprotegerin (OPG) [7]. RANKL is a crucial activator of osteoclastogenesis [8]. In addition, RA serum and synovial fluid present a cytokine profile, including interleukin (IL)1β, IL6, IL17 and tumor necrosis factor (TNF), which further favors osteoclast differentiation and activation since the early phase of the disease [9–11].

Bone is a dynamic tissue organically composed mainly by type I collagen matrix that constitutes the scaffold for calcium hydroxyapatite crystal deposition. Remodeling of bone is a continuous process by which osteoclasts resorb bone tissue and osteoblasts produce new bone matrix that is subsequently mineralized. In this process biochemical markers of bone turnover are produced and released into circulation, providing a read-out of remodeling kinetics. Evidence suggests that bone-remodeling disturbances in RA contribute not only to local bone erosions but also to the development of systemic osteoporosis [12].

We have previously found in the adjuvant-induced rat model of arthritis (AIA) that 22 days of sustained and established inflammatory disease progression directly leads to the degradation of bone biomechanical properties, namely stiffness, ductility and bone strength, which was paralleled by a high collagen bone turnover [13].

Our previous results suggested that the impact of inflammation on bone macro and micro properties occurs very early in arthritis. We now hypothesize that this process starts upon the first inflammatory manifestations and that it directly affects intrinsic bone tissue properties (bone nano properties) [9–11].

The main goal of this work was to analyze the effects of the early phase of systemic inflammatory process at bone tissue level, including nanomechanical properties and microarchitecture.

## Methods

### Animal experimental design

Eighty-eight, 8 week-old female Wistar Han AIA rats weighing approximately 230-250gr were housed in European type II standard filter top cages (Tecniplast, Buguggiate, Italy) and transferred into the SPF animal facility at the Instituto de Medicina Molecular, under a 14h light/10h dark light cycle, acclimatized to T = 20–22°C and RH = 50–60%. They were given access to autoclaved rodent breeder chow (Special Diet Service, RM3) and triple filtered water. AIA rats were purchased from Charles River laboratories international (Barcelona, Spain) and arthritis was induced in their laboratories in 47 animals. The transport service takes 3 days to arrive at Instituto de Medicina Molecular.

Upon arrival, animals were randomly housed in two groups, individually identified and cages were labelled according to the experimental groups, as follows: adjuvant induced arthritis model (N = 47) and control healthy group (N = 41). The inflammatory score, ankle perimeter and body weight were daily evaluated, during disease development. Inflammatory signs were evaluated by counting the score of each joint in a scale of 0–3 (0 –absence; 1 –erythema; 2 –erythema and swelling; 3 –deformities and functional impairment). The total score of each animal was defined as the sum of the partial scores of each affected joint.

Rats were sacrificed at day 11 (healthy control N = 11 and arthritic animals N = 16) and 22 (healthy control N = 30 and arthritic animals N = 31) post disease induction by CO2 narcosis and blood, femurs, tibias and paw samples were collected. In the AIA rat model of arthritis, day 11 post disease induction represents the fast raising phase of polyarticular involvement. Maximum disease activity and severity occurs at day 19 and plateaus up to day 22 post disease induction.[14] In addition, to minimize animal discomfort paper shavings were used as bedding material in Double Decker GR1800 cages (Techniplast, UK) with 5 animals per cage.

All experiments were approved by the Animal User and Ethical Committees at the Instituto de Medicina Molecular (Lisbon University), according to the Portuguese law and the European recommendations, Directive 2010/63/EU revising Directive 86/609/EEC.

### Histological evaluation of hind paws

Histology was performed to evaluate the effect of inflammation on articular joint synovium and bone structures.

Left hind paw samples collected at the time of sacrifice were fixed immediately in 10% neutral buffered formalin solution and then decalcified in 10% formic acid. Samples were then dehydrated and embedded in paraffin, serially sectioned at a thickness of 5μm. Sections were stained with hematoxylin and eosin for histopathological evaluation of structural changes and cellular infiltration. This evaluation was performed in a blind fashion using 5 semi-quantitative scores:

- Sublining layer infiltration score (0-none to diffuse infiltration; 1-lymphoid cell aggregate; 2-lymphoid follicles; 3-lymphoid follicles with germinal center formation);
- Lining layer cell number score (0-fewer than three layers; 1-three to four layers; 2-five to six layers; 3-more than six layers);
- Bone erosion score (0-no erosions; 1-minimal; 2-mild; 3-moderate; 4-severe);
- Cartilage surface (0 –normal; 1 –irregular; 2 –clefts; 3 –clefts to bone);
- Global severity score (0-no signs of inflammation; 1-mild; 2-moderate; 3-severe) [15].

Images were acquired using a Leica DM2500 (Leica Microsystems, Wetzlar, Germany) microscope equipped with a color camera.

### Biomarkers quantification

Serum samples were collected at the sacrifice time and stored at -80°C.

IL-6 is one of the most powerful proinflammatory cytokines and is a key signal for bone destruction. IL-6 was quantified in serum samples using a specific rat ELISA kit (Boster Bio, California, USA). Bone resorption marker CTX I reflects osteoclastic activity as it is a degradation product of type I collagen, the major structural protein of bone. The bone formation marker P1NP is a bio product of type I collagen synthesis and thus is a marker for osteoblastic activity. Bone remodeling markers, CTX-I and P1NP, were quantified by Serum Rat Laps ELISA assay (Immunodiagnostic Systems Ltd, Boldon, UK) in order to study the effects of inflammation on bone turnover.

For all biomarkers standard curves were generated by using reference biomarker concentrations supplied by the manufacturers. Samples were analyzed using a plate reader Infinite M200 (Tecan, Mannedorf, Switzerland).

### Micro-computed tomography (micro-CT) analysis

Structural properties of the trabecular and cortical tibiae were determined with a high-resolution micro-CT system (SkyScan 1272, Bruker microCT, Kontich, Belgium) in order to study the effects of inflammation on bone microstructure. Moist bones were wrapped in parafilm and covered with dental wax to prevent drying and movement during the scanning. X-ray tube was set to 50kV and beam was filtered with 0.5mm Aluminum filter. Sample position and camera settings were tuned to provide 3.0μm isotropic pixel size and projection images were collected every 0.2°. Tissue mineral density values were calibrated against hydroxyapatite phantoms with densities of 250mg/cm^3^ and 750mg/cm^3^. Reconstructions were done with NRecon (v 1.6.9.8; Bruker microCT, Kontich, Belgium) where appropriate corrections to reduce beam hardening and ring artifacts were applied. Bone was segmented in slices of 3μm thickness. After 200 slices from growth plate, we selected and analyzed 1400 slices of trabecular bone. For cortical bone 300 slices (1800 slices from growth plate) were analyzed.

This evaluation was performed in agreement with guidelines for assessment of bone microstructure in rodents using micro-computed tomography [16]. Trabecular bone morphology was analyzed by applying global threshold and despeckle to provide binary image for 3D analyzes. For cortical bone ROI was refined with ROI-shrink wrap operation. This was followed by segmentation of blood vessels using adaptive thresholding. Blood vessels and porosity were analyzed using 3D morphological analyses.

### Bone mechanical tests

Flexural tests were used to evaluate the impact of early inflammation on whole bone mechanical competence of arthritic and healthy controls at day 11 and 22 post disease induction. Femurs were subjected to a 3-point bending test using the universal testing machine (Instron 3366, Instron Corp., Massachusetts, USA). Femurs were placed horizontally anterior side upwards on a support with span length of 5mm. The load was applied with a constant speed of 0.005mm/s until failure occurred. Stiffness was analyzed by fitting first-degree polynomial function to the linear part of recorded load deformation data. A displacement of 0.15μm between fitted slope and measured curve was used as criteria for yield point, whereas the breaking point was defined as set where force reached maximal value. For both yield and breaking points, force, deformation and absorbed energy were defined.

### Nanoindentation

Nanoindentation was performed using a CSM-Nano Hardness Tester System (CSM Instruments SA; Switzerland; Indentation v.3.83) equipped with a Berkovich based pyramid diamond indenter in order to evaluate the impact of early inflammation on nano-mechanical properties of bone tissue in arthritic and healthy controls at day 11 and 22 post disease induction. After micro-CT, 0.5mm of the proximal extremity of tibia was discarded and the remaining tibia was embedded to low viscosity epoxy resin (EpoThin, Buehler, Knorring Oy Ab, Helsinki, Finland). Slow speed diamond saw was used to remove 10% of total bone length. The sample surface was polished using silicon carbide sandpaper with a decreasing grid size (800, 1200, 2400 and 4800) and finished with cloth containing 0.05μm γ-alumina particles. Indentation protocol was adopted from previous work [17] and an average of 8 indentations were done on a transversal section of cortical and trabecular bone with quasi-static (so called ‘advanced’) loading protocol. All indentation regions were selected under an optical microscope to achieve the precise location of indentations. For cortical bone, indentations were placed uniformly through the central cortex and were evenly spaced along bone cross section, avoiding osteocyte lacunae and vascular canals. Our goal was to determine the average properties at mid -diaphysis rather than properties at certain specific locations [18].

In the ‘advanced’ protocol, a trapezoidal loading waveform was applied with a loading/unloading rate of 20mN/min, and with an intermediate load-hold-phase lasting 30s hold at a maximum load 10mN. The approximate depth range of indentations varied between 1.2 up to 3.5μm. The hardness (H_IT_), indentation modulus (E_IT_), indentation creep (C_IT_) and elastic part of indentation work (η_IT_) were measured by advanced protocol using the Oliver and Pharr (1992) method [19].

Histological images of rat tibiae from diaphyseal cortical region were acquired during the nanoindentation technique, using a CSM instruments (Switzerland) microscope equipped with a color camera.

A histologic score was applied in order to evaluate the lamellar structures of bone tissue. This evaluation was performed in a blind fashion using a semi-quantitative score:

- Lamellar bone structure: (1- predominantly parallel-lamella; 2—concentric and parallel-lamellae in the same proportion; 3 –predominantly concentric lamella).

The ratio of osteocyte lacuna area / total tissue area was also evaluated at x200 magnification in order to analyse the percentage of total tissue area occupied by osteocyte lacunae. The method of acquisition and analysis used was the same applied for the evaluation of bone volume / tissue volume in histomorphometry technique [13]. All variables were expressed and calculated according to the recommendations of the American Society for Bone and Mineral Research [20], using a morphometric program (Image J 1.46R with plugin Bone J).

### Statistical analysis

Statistical differences were determined with Mann–Whitney tests using GraphPad Prism (GraphPad, California, USA). The normality distribution was assessed by D’Agostino and Pearson test. Data are expressed as median with interquartile range. Differences were considered statistically significant for *p<0.05.

## Results

### The AIA rat model has a rapid and severe disease progression

**Fig 1:**
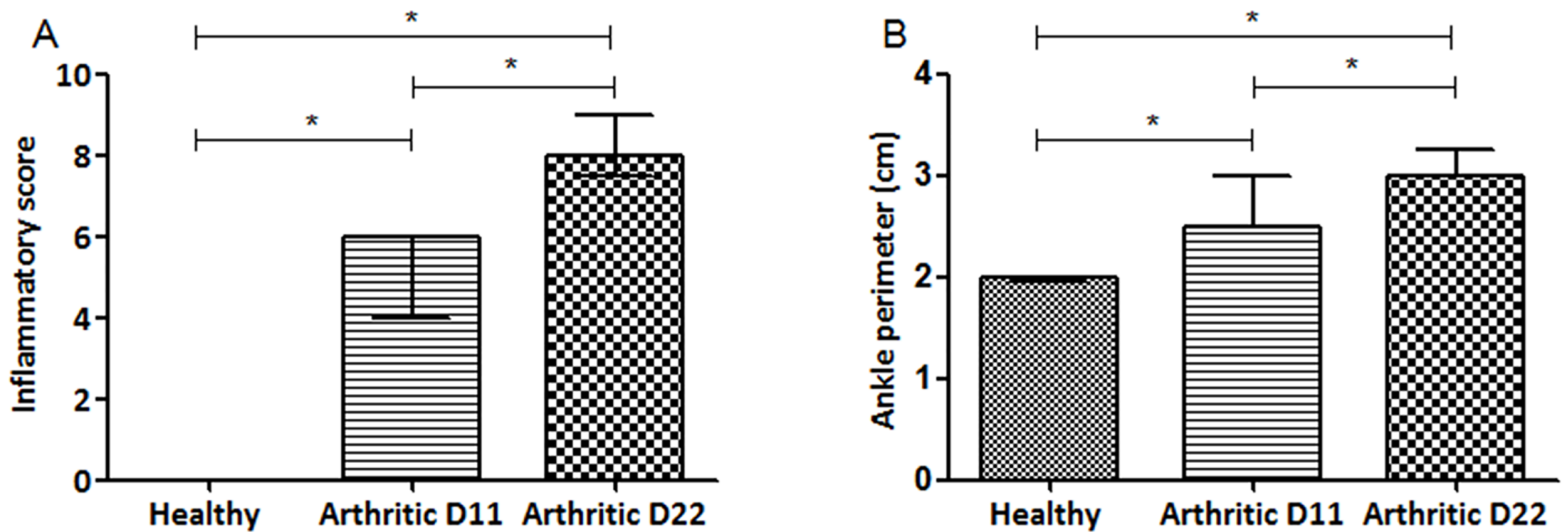
Inflammatory score and ankle perimeter. Arthritic rats have a rapidly disease progression including ankle swelling, when compared with healthy control rats. Statistical differences were determined with non-parametric Mann Whitney test using GraphPad Prism (GraphPad, California, USA). Differences were considered statistically significant for p values ≤ 0.05. Healthy D11 N = 11, Healthy D22 N = 30, Arthritic D11 N = 16 and Arthritic D22 N = 31.

Results showed that inflammatory signs (Fig 1) boosted sharply in the arthritic group. The inflammatory score (Fig 1a) increased significantly at day 11 and 22 post disease induction (which correspond to an acute phase and a chronic phase of systemic inflammation, respectively) in arthritic rats when compared to healthy controls (p<0.0001, respectively).

Moreover, arthritic animals at day 11 and 22 post disease induction sharply increased the ankle swelling throughout disease progression (Fig 1b), when compared to healthy rats (p<0.0001, respectively)

### Inflammation affects local joints and promotes bone damage in AIA rats since the early stage of arthritis

**Fig 2:**
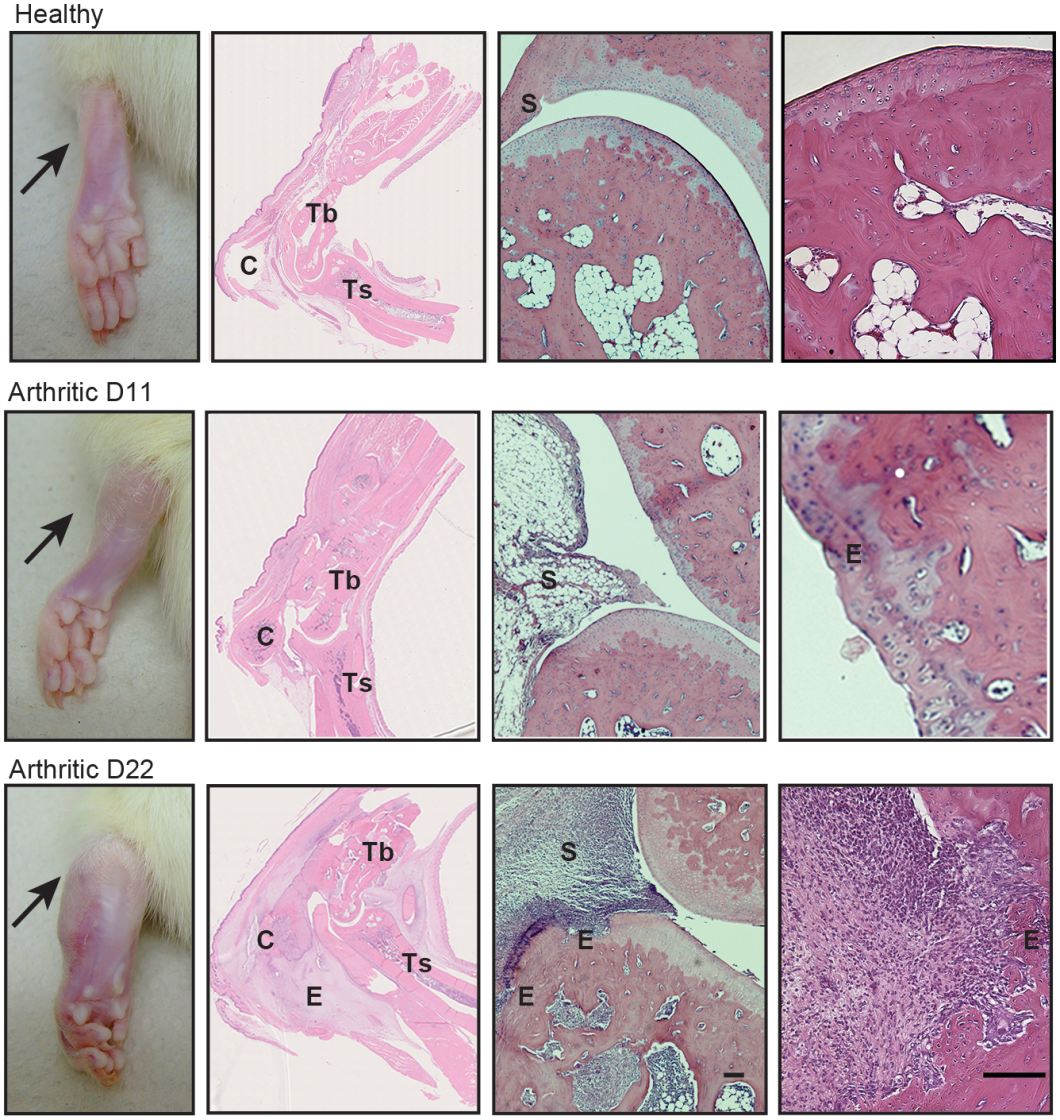
Histological images of joints after 11 and 22 days of disease induction. These patterns are merely illustrative of the type of histological features observed. Black arrow indicates the absence/presence of ankle swelling in rat hind paws. C–calcaneus, E–edema or erosion, S–synovia, Tb–tibia, Ts–tarso. Magnification of 50X. Bar: 100 μm.

To evaluate the effect of inflammation in local articular joint synovium and bone structures, paw sections stained with hematoxylin and eosin were performed (illustrative images can be observed in Fig 2).

**Fig 3:**
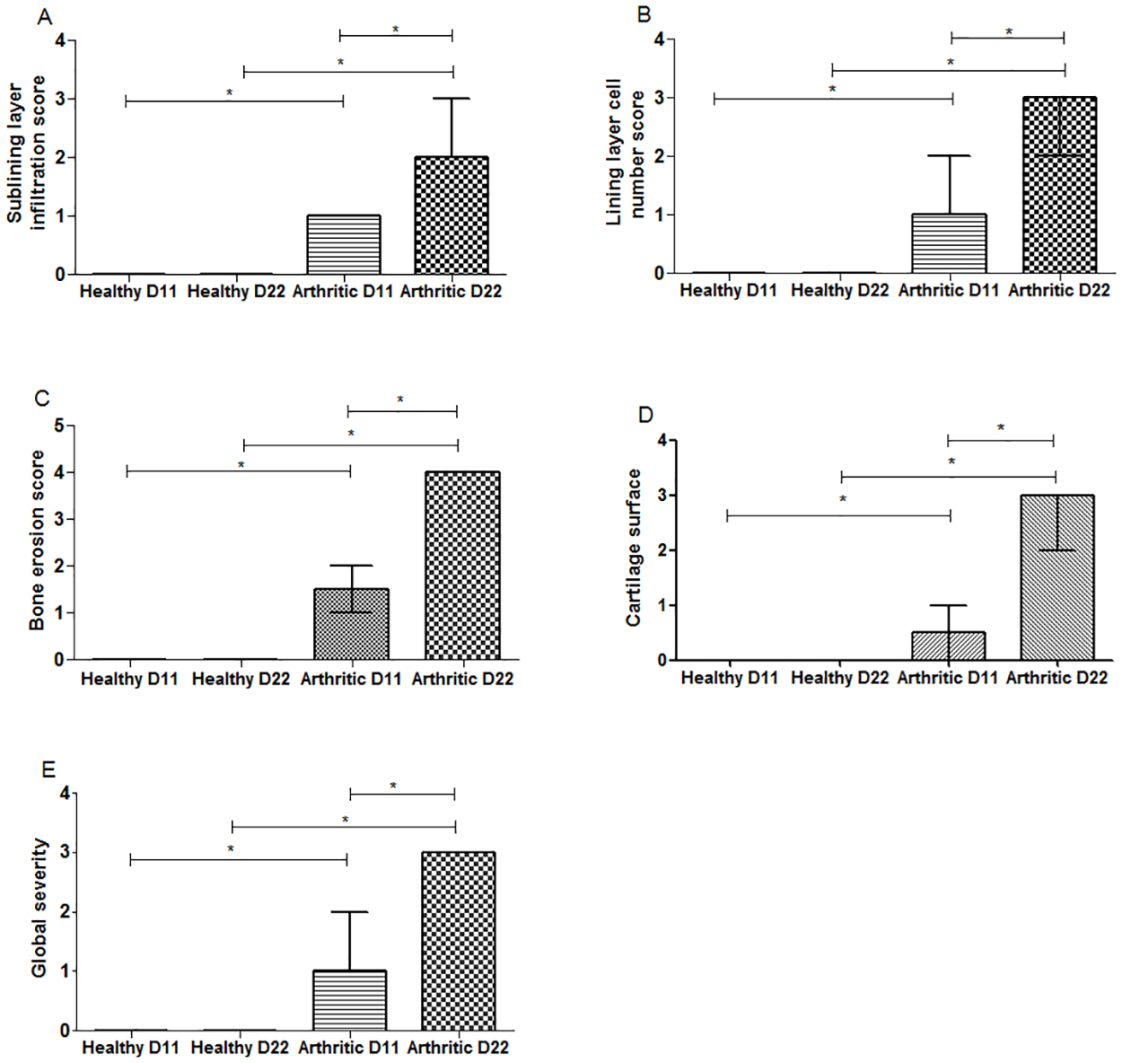
Semi-quantitative evaluation of histological sections of inflammation and tissue damage locally in the joints of AIA rats. Notice that results demonstrate that arthritic rats after 11 and 22 days of disease induction increase cellular infiltration (A), number of lining layer cells (B), bone erosions (C) and cartilage surface damage (D). Global disease severity demonstrates this marked inflammation and progression between day 11 and 22 (E). Data are expressed as median with interquartile range. Differences were considered statistically significant for p-values<0.05, according to the Mann Whitney test. Healthy D11 N = 11, Healthy D22 N = 30, Arthritic D11 N = 16 and Arthritic D22 N = 31.

The histological evaluation using 5 semi-quantitative scores is depicted in Fig 3.

Sublining layer infiltration (Fig 3a), number of lining layer cells (Fig 3b) and bone erosion score (Fig 3c) were increased in the arthritic group when compared with healthy controls at day 11 and 22 post disease induction (p<0.0001). Arthritic samples also showed increased cartilage damage surface (Fig 3d) since the early phase of arthritis at day 11 and 22 (p = 0.0403 and p<0.0001 vs healthy controls, respectively). These data contributed to the increased values of severity score (Fig 3e) in arthritic group (p<0.0001 vs healthy controls). Moreover, results also demonstrated a continuous disease progression between day 11 and 22 in arthritic animals, as observed by the increase of the sublining layer infiltration, number of lining layer cells, bone erosion score (p<0.0001), cartilage surface score (p = 0.0001) and global severity score (p = 0.0006).

### Systemic inflammation occurs in this model

**Fig 4:**
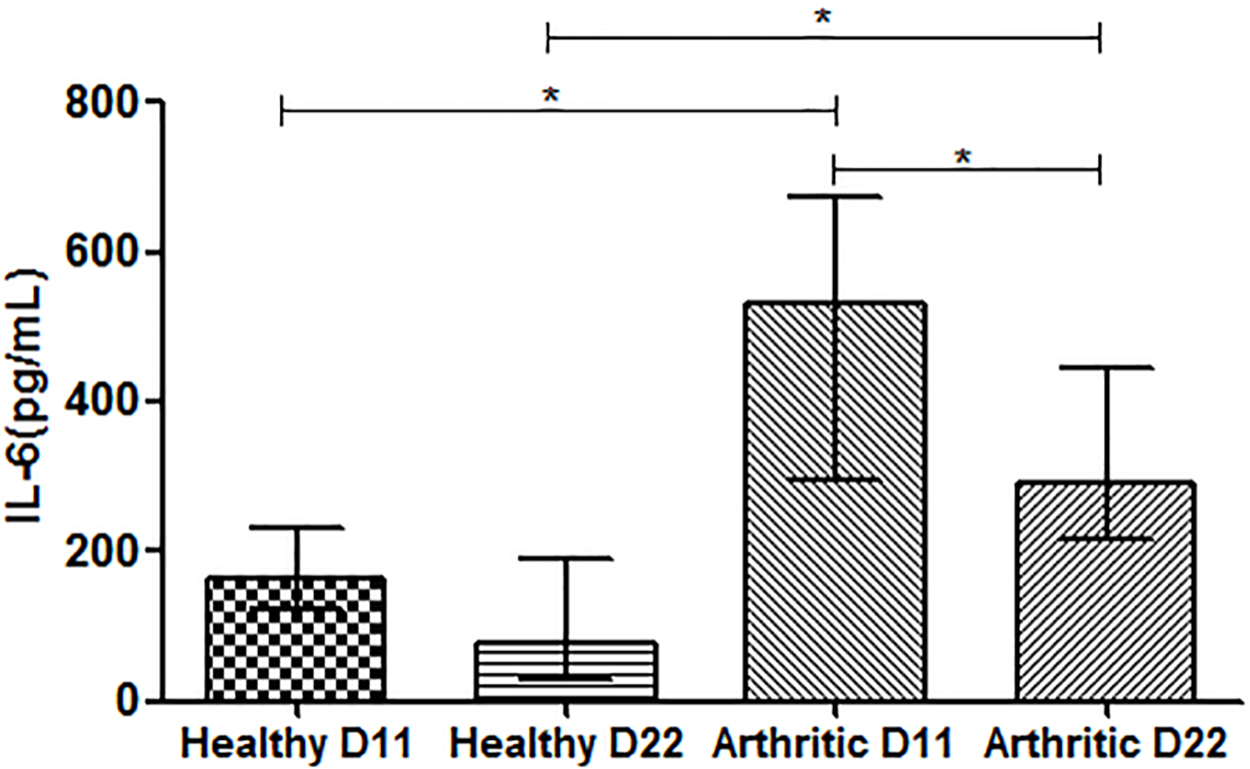
Serum quantification of IL6. Serum samples collected at day 11 and 22 post disease induction were analyzed by ELISA technique. IL6 was increased in arthritic rats at day 11 and 22 (p = 0.0003 and p<0.0001vs healthy controls, respectively). Differences were considered statistically significant for p-values<0.05, according to the Mann–Whitney tests Healthy D11 N = 11, Healthy D22 N = 21, Arthritic D11 N = 16 and Arthritic D22 N = 23.

We observed that IL6 levels were increased in the serum of arthritic rats at day 11 and 22 post disease induction in comparison with healthy controls (p = 0.0003 and p<0.0001, respectively), as observed in Fig 4. Results also revealed that IL6 levels decreased in arthritic rats at day 22 when compared with day 11 (p = 0.0092).

### Systemic inflammation promotes high bone turnover

**Fig 5:**
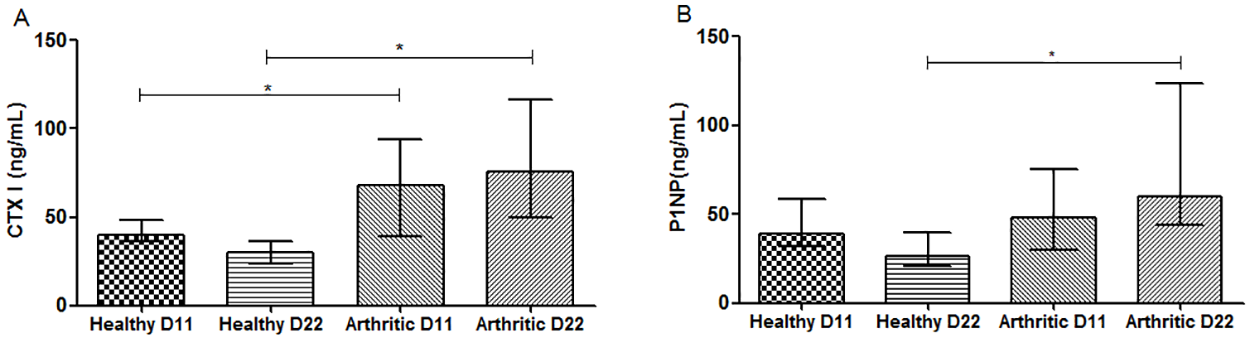
Bone turnover markers quantification. Serum samples collected at day11 and 22 post disease induction were analyzed by ELISA technique. Bone resorption marker, CTX-I (A) and bone formation marker, P1NP (B) were increased in arthritic rats at day 22 (p<0.0001 and p = 0.0007, respectively). Results also demonstrate increased values of CTX-I in arthritic rats at day 11 when compared with healthy controls (p = 0.0218). Differences were considered statistically significant for p-values<0.05, according to the Mann–Whitney tests. Healthy D11 N = 11, Healthy D22 N = 18, Arthritic D11 N = 16 and Arthritic D22 N = 18.

We have observed that both CTX-I (Fig 5a) and P1NP (Fig 5b) were significantly increased in the arthritic group at day 22 in comparison with healthy controls (p<0.0001 and p = 0.0007, respectively), revealing an increase of bone turnover in the arthritic group. Moreover, arthritic rats showed already increased values of CTX-I at day 11 post disease induction (p = 0.0218 vs healthy rats at day 11) but not of P1NP. These results suggest that systemic inflammation promotes skeletal bone turnover disturbances since the early stages of arthritis.

### Micro-CT

The effect of systemic inflammation on cortical and trabecular skeletal bone was assessed by micro-CT in bone tibia.

**Fig 6:**
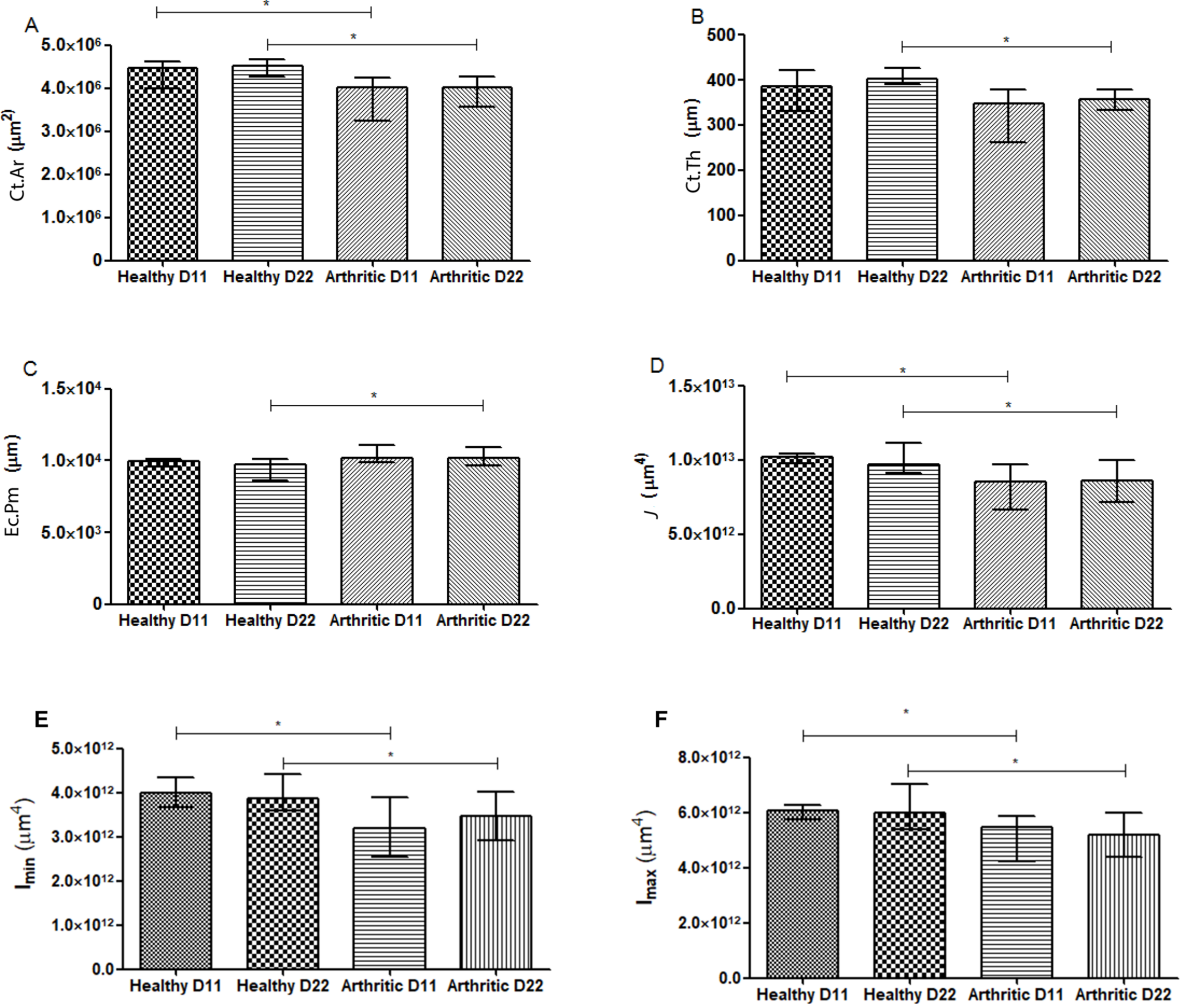
Micro-computed tomography (micro-CT)—Cortical analysis of tibiae rat sample. The cortical bone area showed decreased values in the arthritic group at day 11 and 22(A), as well as the polar moment of inertia (D), the minimum (Imin) (mediolateral) (E) and the maximum (Imax) (anteroposterior) (F) moment of inertia. Arthritic group at day 22 presented a marked deterioration of bone tibia demonstrated by decreased average cortical thickness (B) and increased endocortical perimeter (C). Differences were considered statistically significant for p-values<0.05, according to the Mann–Whitney tests. Healthy D11 N = 11, Healthy D22 N = 30, Arthritic D11 N = 16 and Arthritic D22 N = 31.

The arthritic group showed at day 22 a dramatic deterioration of bone tibia integrity associated with a reduction in cortical bone area (Fig 6a) and average cortical thickness (Fig 6b) (p<0.0001 vs healthy controls, respectively) with an evident increased endocortical perimeter (Fig 6c) (p = 0.0029 vs healthy control). However, changes promoted by inflammation on bone structure begin at the early stages of arthritis as we can observe by the results obtained in the arthritic group by day 11 with a decreased cortical bone area (Fig 6a) (p = 0.0219 vs healthy control). Moreover, polar moment of inertia (Fig 6d) (p = 0.0091 and p = 0.0024) and both minimum (Imin) (Fig 6e) (p = 0.0146 and p = 0.0170) and maximum (Imax) (Fig 6f) (p = 0.0406 and p = 0.0012) were decreased in arthritic animals at day 11 and 22 post disease induction, when compared to the respective healthy control.

**Fig 7:**
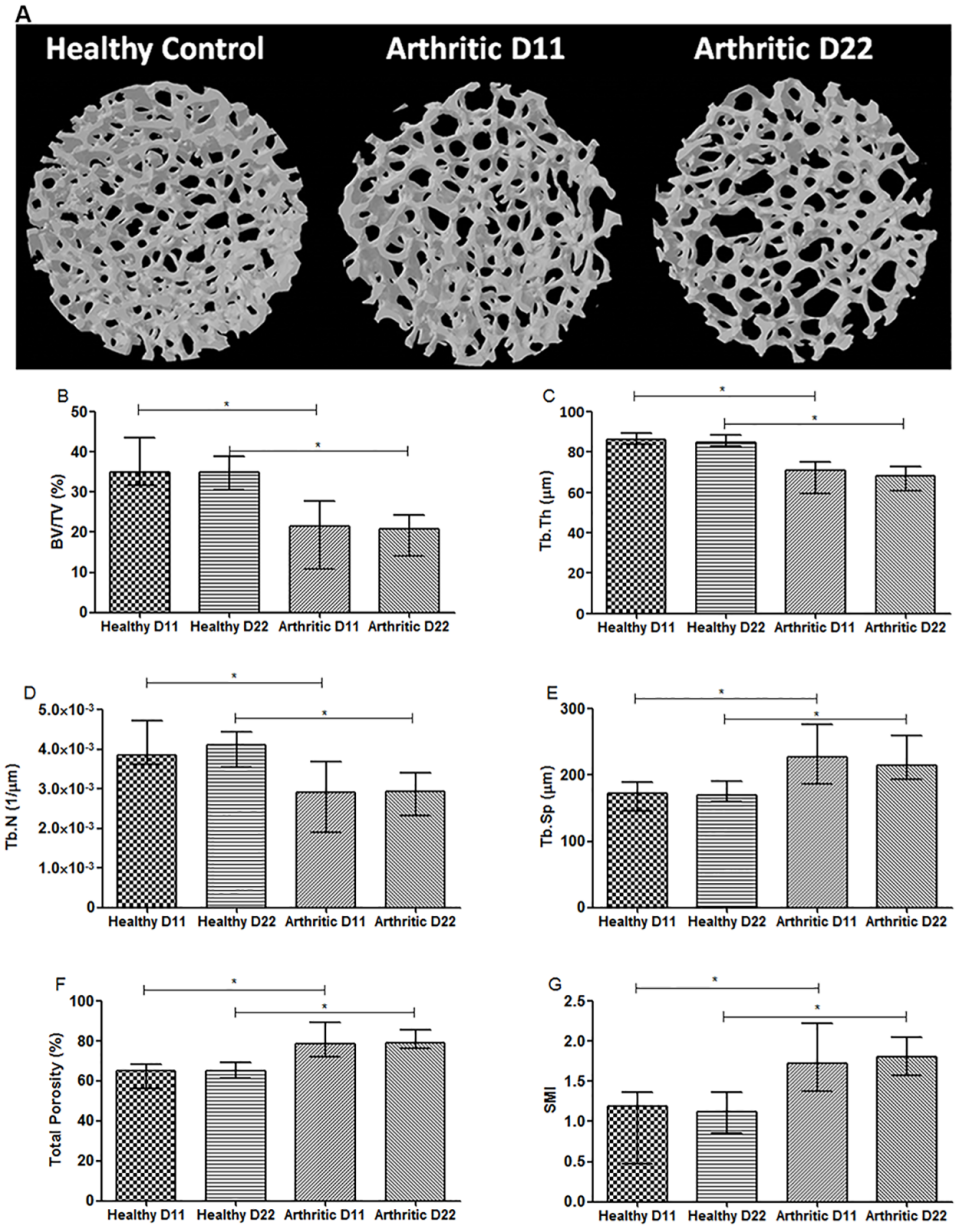
Micro-computed tomography (micro-CT)—Trabecular analysis of tibiae rat sample. MicroCT images from healthy and arthritic tibiae rats (A). Images acquired with SkyScan 1272, Bruker microCT, Belgium. Results showed decreased values of the ratio bone volume/tissue volume (B), trabecular thickness (C) and number (D) in arthritic group at day 11 and 22 post disease induction. Trabecular bone also showed increased values of trabecular separation (E), porosity (F) and structural model index in both arthritic groups (G). Differences were considered statistically significant for p-values<0.05, according to the Mann–Whitney tests. Healthy D11 N = 11, Healthy D22 N = 30, Arthritic D11 N = 16 and Arthritic D22 N = 31.

Trabecular bone (Fig 7) also showed increased deterioration promoted by inflammation with decreased trabecular bone volume fraction in arthritic rats at day 11 and 22 post disease induction (Fig 7b) (p = 0.0001 and p<0.0001 vs healthy controls, respectively), thickness (Fig 7c) (p<0.0001 vs healthy controls, respectively), and number (Fig 7d) (p = 0.0039 and p<0.0001 vs healthy controls, respectively). Results also demonstrated increased values of trabecular separation in the arthritic group at day 11 and 22 (Fig 7e) (p = 0.0043 and p<0.0001 vs healthy controls) and of porosity (Fig 7f) (p = 0.0001 and p<0.0001 vs healthy controls, respectively). Furthermore, structure model index (Fig 7g) showed increased values in arthritic groups at day 11 and 22 (p = 0.0015 and p<0.0001 vs healthy controls, respectively) indicating that the shape of trabeculae is rather rod-like in the arthritic group as compared to plate-like shape in healthy controls.

Altogether, these results showed that inflammation promote bone structural disturbances, leading to bone loss and consequent bone fragility in arthritic rats (Fig 7a).

### Mechanical tests

**Fig 8:**
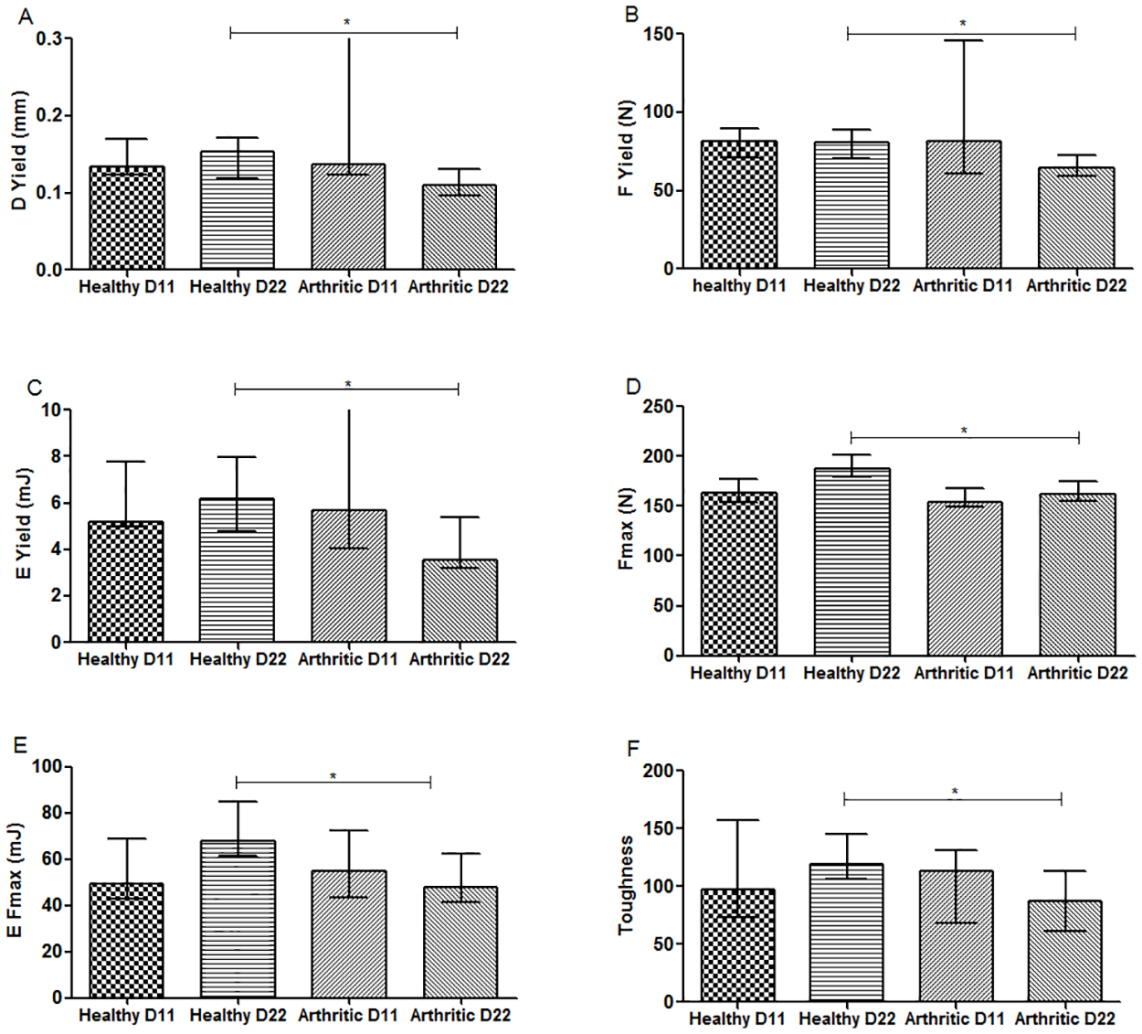
Bone mechanical properties assessed by three-point bending tests in rat femur. Results showed that arthritic rats at day 22 have decreased properties at yield point, related to displacement (A), strength (B) and energy (elastic energy) (C). Arthritic bones at day 22 required a lower maximum load (D) to fracture, with a decreased elastic energy at maximum load (E) and toughness (F). Differences were considered statistically significant for p-values<0.05, according to the Mann–Whitney tests. Healthy D11 N = 5, Healthy D22 N = 14, Arthritic D11 N = 5and Arthritic D22 N = 10.

Classical mechanical properties of rat femurs were evaluated using 3-point bending mechanical tests. The stress at yield point was noted where according to Zioupos et al (1994) the first micro fractures appear and irreversible damage occurs. Another interesting point is maximal load at breaking point (where complete fracture occurs) and toughness can be estimated. As shown in Fig 8, arthritic rats at day 22 revealed biomechanical disturbances with a decrease in mechanical properties at yield point, namely by displacement (Fig 8a) (p = 0.0192 vs healthy control), strength (Fig 8b) (p = 0.0229 vs healthy control) and pre yield energy (Fig 8c) (elastic energy) (p = 0.0161 vs healthy control). These results suggest that arthritic bones at day 22 start to accumulate micro fractures with smaller deformations and loads, leading to a decreased energy absorption capability at yield point. Results also demonstrated that arthritic rats at day 22 have decreased maximum load (Fig 8d) and elastic capabilities at maximum load point (Fig 8e) (p = 0.0017 and p = 0.0134 vs healthy control, respectively), which indicates increased bone fragility. Finally, arthritic rat groups showed a significant decrease in toughness (Fig 8f) (p = 0.0143 vs healthy control), demonstrating that arthritic bone can absorb less energy before fracturing.

Altogether, mechanical data revealed that arthritic groups have significantly lower mechanical properties as compared to healthy controls, meaning that arthritic bones are more fragile and prone to fracture, as highlighted by the significantly lower structural strength and poor biomechanical properties.

### Decreased hardness in arthritic bone associated with an increase of the ratio of bone concentric to parallel lamellae and of the area of the osteocyte lacuna

**Fig 9:**
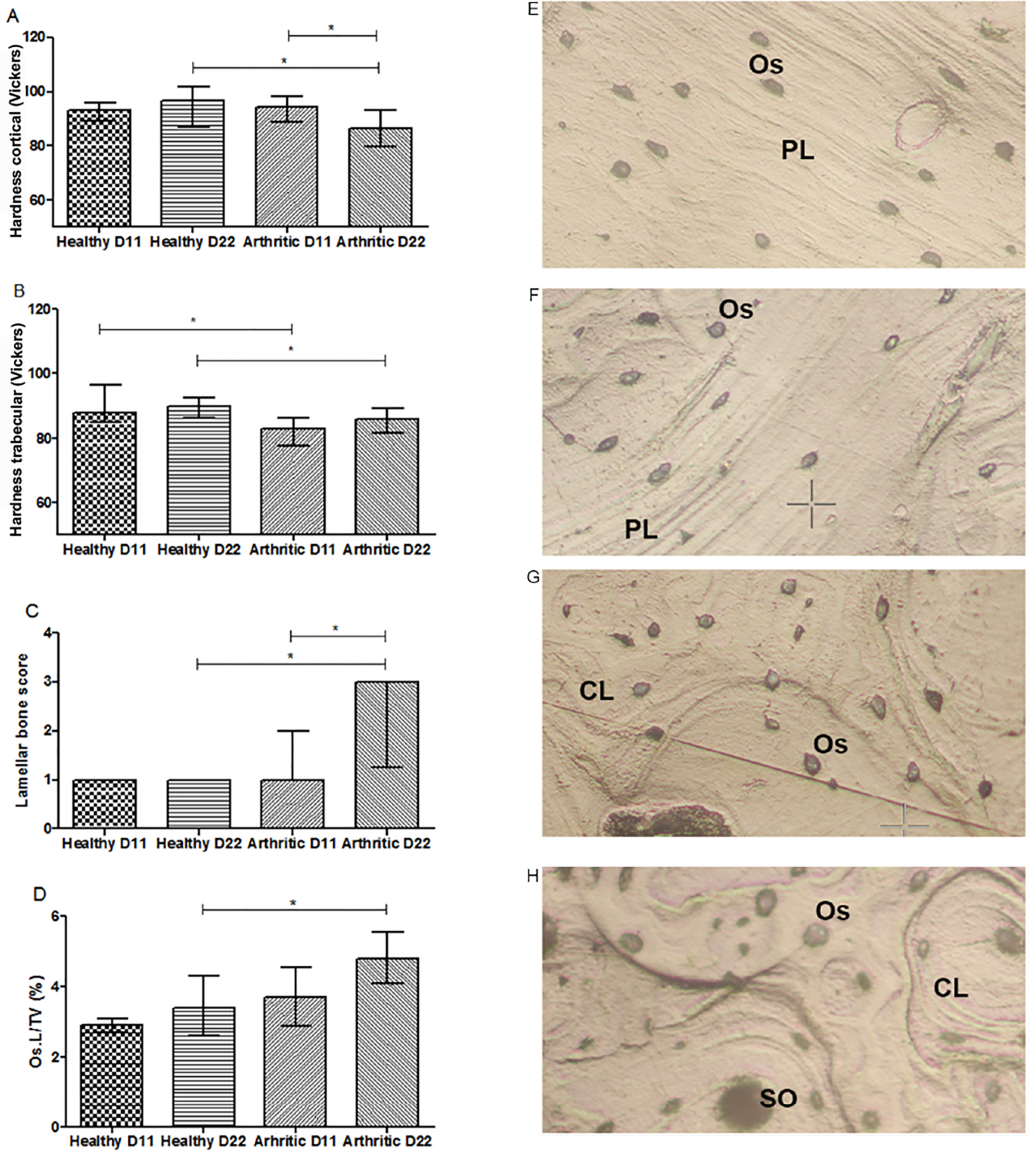
Bone mechanical properties assessed by nanoindentation in rat femur at 11 and 22 days post disease induction and respective optical micrographs from the indentation tissue area. Nano-mechanical tests revealed that arthritic rats have decreased cortical hardness at day 22 and of trabecular hardness at day 11 and 22 post disease induction (B). Results demonstrated that concentric lamellae (C) and ratio of area occupied by osteocyte lacunae in the total tissue (D) are increased when compared to healthy animals at day 22. Images are merely illustrative of the type of histological features observed. Concentric lamellas are identified in secondary osteons (SO), characteristic from arthritic animals at day 11 (G) and 22(H). On the contrary, parallel-lamellae (PL) are identified in healthy at day 11 (E) and 22 (F). Os—Osteocytes, SO—Secondary osteons, PL—Parallel-lamellae, CL—Concentric lamellas. Magnification 20X. Differences were considered statistically significant for p-values<0.05, according to the Mann–Whitney tests. Healthy D11 N = 11, Healthy D22 N = 28, Arthritic D11 N = 16 and Arthritic D22 N = 21.

Nanoindentation was performed in order to assess the quality at tissue matrix level as this technique works at the level of a single trabecula or within a confined submicron area of the cortical bone tissue (Fig 9).

Nano-mechanical tests revealed that arthritic rats have decreased hardness in the cortical aspect of bone at day 22 post disease induction (Fig 9a) (p = 0.0010 vs healthy control) and at trabecular bone at day 11 and 22 post disease induction (Fig 9b) (p = 0.0184 and p = 0.008 vs healthy controls, respectively). Results also demonstrated the continuous decreasing of cortical hardness (Fig 9a) during arthritis development among arthritic groups (p = 0.0043). No differences were observed in the other parameters analysed.

Topographic images gathered during nanoindentation allowed the characterization of histologic features from healthy and arthritic bone at day 11 (Fig 9g) and 22 (Fig 9h) days post disease induction. Concentric lamellas were identified in secondary osteons (SO) and more frequently observed in arthritic animals than in healthy controls (Fig 9c) (p = 0.0022, arthritic group at day 22 vs healthy control rats at day 22). On the contrary, healthy animals at day 11 (Fig 9e) and 22 (Fig 9f) presented more parallel-lamellae (PL) structures than SO structures.

Arthritic animals at day 22 post disease induction showed also an increased area occupied by osteocyte lacunae in the total tissue when compared to healthy animals (p = 0.0067) (Fig 9d). Results also demonstrated a slight tendency towards an increase at day 11 post disease induction (Fig 9d).

## Discussion

Arthritic groups presented inflammatory manifestations with synovial tissue inflammation and local bone erosions, as expected. Increased values of serum IL-6 were observed in arthritic rats since the early stages of arthritis, confirming the systemic inflammatory component of this animal model. This cytokine plays a pivotal role in the pathologic processes of arthritis with a special emphasis on its impact on skeletal bone [21–24]. In accordance with this effect an increased and accelerated bone turnover was shown in arthritic animals, as depicted by increased CTX-I and P1NP levels since the early stages of arthritis. Data already published by our group in the same animal model of arthritis had also shown that P1NP levels were increased at day 22 post disease induction in arthritic animals and so did CTX-I levels[13], reflecting an overall increase in bone turnover [25]. Despite the existing of some variability in human studies, CTX-I and P1NP have been found to be increased in RA patients, revealing the coupled compensatory mechanism of bone turnover [13,26].

Micro-CT confirmed that interference of inflammation with bone metabolism translates into bone micro architectural and geometric changes in trabecular and cortical bone, since the early phase of arthritis– 11 days after disease induction. In addition micro-CT provides mechanical insights such as polar moment of inertia, which represents the material resistance to torsional deformation in cylindrical objects or segments of cylindrical objects, suggesting low mechanical competences in arthritic bone [27]. Because of the uncertainty on the direction of the predominant bending moments in-vivo we elected to analyse the results with respect to the polar moment of inertia. We have also considered the maximal (Imax) and minimal (Imin) second moments of area (area moments of inertia), which arguably should correspond to anteroposterior (IAP) and mediolateral (IML) directions in-vivo although there is no way of proving that.

For micromechanical property analysis we have selected the most reliable nanomechanical parameters, which reflect the material property (S1 Fig).

The three-point bending test confirmed the previous result, demonstrating decreased yield properties and reduced maximum load bearing capacity, which was also reflected in the energy absorption of all mechanical parameters evaluated at 22 days post disease induction. This mechanical fragility, replicating the clinical observations in RA patients, further reinforces the use of the AIA model as an adequate strategy for a fast insight on the impact of inflammation on bone. The first part of this study sets the stage for using this model for evaluating the effects of the early phase of systemic inflammatory process at bone tissue level, including nanomechanical properties and microarchitecture.

Nanoindentation was performed in order to assess the quality of bone at tissue matrix level, as this technique can be used at the level of a single trabecula or within a confined submicron area of the cortical bone tissue. Results showed decreased cortical and trabecular hardness in arthritic rats since the early phase of arthritis (days 11 and 22).

We also observed at day 11 and 22 post arthritis induction concentric lamellas in secondary osteons (SO) microstructures, resulting from high bone remodelling, as previously described [13,28,29]. Dall’Ara et al. suggested that larger numbers of this younger, less mineralised and less hard structures, could be related to reduced hardness of bone tissue identified by nanoindentation. On the contrary, healthy animals presented more parallel-lamellae (PL) structures than SO structures and this PL structures are 10% more harder than the former, representing the mature bone structure (and normal bone remodelling)[29]. In addition, arthritic animals had an increased area occupied by osteocyte lacunae in total tissue. Osteocytes are responsible for the maintenance of the bone homeostasis, regulating the behaviour of osteoblasts and osteoclasts by communicating through gap junctions [30]. Although no previous data is available in the context of arthritis some studies revealed that osteocytes from osteoarthritis patients have an irregular morphology, with limited ability to reply to mechanical stimuli, leading to significant changes in the structure and mineral density [31]. Despite being still unclear this apparent change of osteocyte morphology in arthritic bone might contribute to the structural and mechanical changes observed in this context.

These results are in line with our previous results where we also demonstrated that inflammation induces bone mineral loss since the early phase of arthritis [13]. Other publications from our group in a chronic model of arthritis, showed a decreased mineral content [13] and also a lower density and organization of collagen fibrils when compared to healthy control bone [32].

## Conclusion

Systemic inflammation induces very early changes in bone microarchitecture and macro and mechanical behaviour leading to increased bone fragility. At a tissue level a decreased tissue hardness is observed, associated with changes in bone lamella organization and osteocyte lacuna surface.

## Supporting information

S1 Fig 3D model of the mouse tibia cross section showing a triangularly shaped bone. (TIF)

## References

[1] Gibofsky A (2012) Overview of epidemiology, pathophysiology, and diagnosis of rheumatoid arthritis. Am J Manag Care 18: S295–302. 23327517

[2] Yelin E, Callahan LF (1995) The economic cost and social and psychological impact of musculoskeletal conditions. National Arthritis Data Work Groups. Arthritis Rheum 38: 1351–1362. 7575685 10.1002/art.1780381002

[3] Lin YY, Jean YH, Lee HP, Chen WF, Sun YM, et al (2013) A soft coral-derived compound, 11-epi-sinulariolide acetate suppresses inflammatory response and bone destruction in adjuvant-induced arthritis. PLoS One 8: e62926 doi: 10.1371/journal.pone.0062926 23675440 10.1371/journal.pone.0062926 PMC3652811

[4] Haugeberg G, Orstavik RE, Uhlig T, Falch JA, Halse JI, et al (2002) Bone loss in patients with rheumatoid arthritis: results from a population-based cohort of 366 patients followed up for two years. Arthritis Rheum 46: 1720–1728. doi: 10.1002/art.10408 12124854 10.1002/art.10408

[5] Marshall D, Johnell O, Wedel H (1996) Meta-analysis of how well measures of bone mineral density predict occurrence of osteoporotic fractures. BMJ 312: 1254–1259. 8634613 10.1136/bmj.312.7041.1254 PMC2351094

[6] Eric-Jan JA K (2000) Bone mass in rheumatoid arthritis. CLINICAL AND EXPERIMENTAL RHEUMATOLOGY.

[7] Fonseca JE, Cortez-Dias N, Francisco A, Sobral M, Canhao H, et al (2005) Inflammatory cell infiltrate and RANKL/OPG expression in rheumatoid synovium: comparison with other inflammatory arthropathies and correlation with outcome. Clin Exp Rheumatol 23: 185–192. 15895888

[8] Boyle WJ, Simonet WS, Lacey DL (2003) Osteoclast differentiation and activation. Nature 423: 337–342. doi: 10.1038/nature01658 12748652 10.1038/nature01658

[9] Moura RA, Cascao R, Perpetuo I, Canhao H, Vieira-Sousa E, et al (2011) Cytokine pattern in very early rheumatoid arthritis favours B-cell activation and survival. Rheumatology (Oxford) 50: 278–282. 21047805 10.1093/rheumatology/keq338

[10] Cascao R, Moura RA, Perpetuo I, Canhao H, Vieira-Sousa E, et al (2010) Identification of a cytokine network sustaining neutrophil and Th17 activation in untreated early rheumatoid arthritis. Arthritis Res Ther 12: R196 doi: 10.1186/ar3168 20961415 10.1186/ar3168 PMC2991033

[11] Caetano-Lopes J, Canhao H, Fonseca JE (2009) Osteoimmunology—the hidden immune regulation of bone. Autoimmun Rev 8: 250–255. doi: 10.1016/j.autrev.2008.07.038 18722561 10.1016/j.autrev.2008.07.038

[12] Caetano-Lopes J, Rodrigues A, Lopes A, Vale AC, Pitts-Kiefer MA, et al (2014) Rheumatoid arthritis bone fragility is associated with upregulation of IL17 and DKK1 gene expression. Clin Rev Allergy Immunol 47: 38–45. doi: 10.1007/s12016-013-8366-y 23546988 10.1007/s12016-013-8366-y

[13] Vidal B, Cascao R, Vale AC, Cavaleiro I, Vaz MF, et al (2015) Arthritis induces early bone high turnover, structural degradation and mechanical weakness. PLoS One 10: e0117100 doi: 10.1371/journal.pone.0117100 25617902 10.1371/journal.pone.0117100 PMC4305284

[14] Lisa R. Schopf KA, Bruce D. Jaffee (2006) In Vivo Models of Inflammation. Basel/Switzerland: Birkhäuser Basel.

[15] Cascao R, Vidal B, Raquel H, Neves-Costa A, Figueiredo N, et al (2012) Effective treatment of rat adjuvant-induced arthritis by celastrol. Autoimmun Rev 11: 856–862. doi: 10.1016/j.autrev.2012.02.022 22415021 10.1016/j.autrev.2012.02.022 PMC3582326

[16] Bouxsein ML, Boyd SK, Christiansen BA, Guldberg RE, Jepsen KJ, et al (2010) Guidelines for assessment of bone microstructure in rodents using micro-computed tomography. J Bone Miner Res 25: 1468–1486. doi: 10.1002/jbmr.141 20533309 10.1002/jbmr.141

[17] Herlin M, Finnila MA, Zioupos P, Aula A, Risteli J, et al (2013) New insights to the role of aryl hydrocarbon receptor in bone phenotype and in dioxin-induced modulation of bone microarchitecture and material properties. Toxicol Appl Pharmacol 273: 219–226. doi: 10.1016/j.taap.2013.09.002 24035824 10.1016/j.taap.2013.09.002

[18] Zhang R, Gong H, Zhu D, Ma R, Fang J, et al (2015) Multi-level femoral morphology and mechanical properties of rats of different ages. Bone 76: 76–87. doi: 10.1016/j.bone.2015.03.022 25857690 10.1016/j.bone.2015.03.022

[19] W.C. Oliver GMP (1992) An improved technique for determining hardness and elastic modulus using load and displacement sensing indentation experiments.

[20] Parfitt AM, Drezner MK, Glorieux FH, Kanis JA, Malluche H, et al. (1987) Bone histomorphometry: standardization of nomenclature, symbols, and units. Report of the ASBMR Histomorphometry Nomenclature Committee. J Bone Miner Res 2: 595–610. 10.1002/jbmr.5650020617 3455637

[21] Choy EH, Isenberg DA, Garrood T, Farrow S, Ioannou Y, et al (2002) Therapeutic benefit of blocking interleukin-6 activity with an anti-interleukin-6 receptor monoclonal antibody in rheumatoid arthritis: a randomized, double-blind, placebo-controlled, dose-escalation trial. Arthritis Rheum 46: 3143–3150. doi: 10.1002/art.10623 12483717 10.1002/art.10623

[22] Nishimoto N, Yoshizaki K, Miyasaka N, Yamamoto K, Kawai S, et al (2004) Treatment of rheumatoid arthritis with humanized anti-interleukin-6 receptor antibody: a multicenter, double-blind, placebo-controlled trial. Arthritis Rheum 50: 1761–1769. doi: 10.1002/art.20303 15188351 10.1002/art.20303

[23] Maeshima K, Yamaoka K, Kubo S, Nakano K, Iwata S, et al (2012) The JAK inhibitor tofacitinib regulates synovitis through inhibition of interferon-gamma and interleukin-17 production by human CD4+ T cells. Arthritis Rheum 64: 1790–1798. doi: 10.1002/art.34329 22147632 10.1002/art.34329

[24] Fonseca JE, Santos MJ, Canhao H, Choy E (2009) Interleukin-6 as a key player in systemic inflammation and joint destruction. Autoimmun Rev 8: 538–542. doi: 10.1016/j.autrev.2009.01.012 19189867 10.1016/j.autrev.2009.01.012

[25] Siebuhr AS, Wang J, Karsdal M, Bay-Jensen AC, Y J, et al (2012) Matrix metalloproteinase-dependent turnover of cartilage, synovial membrane, and connective tissue is elevated in rats with collagen induced arthritis. J Transl Med 10: 195 doi: 10.1186/1479-5876-10-195 22992383 10.1186/1479-5876-10-195 PMC3551788

[26] Cortet B, Flipo RM, Pigny P, Duquesnoy B, Boersma A, et al (1998) Is bone turnover a determinant of bone mass in rheumatoid arthritis? J Rheumatol 25: 2339–2344. 9858427

[27] Ugural A, Fenster S (1995) Advanced Strength and Applied Elasticity: Prentice-Hall Inc.

[28] Bailey AJ, Mansell JP, Sims TJ, Banse X (2004) Biochemical and mechanical properties of subchondral bone in osteoarthritis. Biorheology 41: 349–358. 15299267

[29] Dall'Ara E, Ohman C, Baleani M, Viceconti M (2011) Reduced tissue hardness of trabecular bone is associated with severe osteoarthritis. J Biomech 44: 1593–1598. doi: 10.1016/j.jbiomech.2010.12.022 21496822 10.1016/j.jbiomech.2010.12.022

[30] Taylor AF, Saunders MM, Shingle DL, Cimbala JM, Zhou Z, et al (2007) Mechanically stimulated osteocytes regulate osteoblastic activity via gap junctions. Am J Physiol Cell Physiol 292: C545–552. doi: 10.1152/ajpcell.00611.2005 16885390 10.1152/ajpcell.00611.2005

[31] Jaiprakash A, Prasadam I, Feng JQ, Liu Y, Crawford R, et al (2012) Phenotypic characterization of osteoarthritic osteocytes from the sclerotic zones: a possible pathological role in subchondral bone sclerosis. Int J Biol Sci 8: 406–417. doi: 10.7150/ijbs.4221 22419886 10.7150/ijbs.4221 PMC3303142

[32] Caetano-Lopes J, Nery AM, Canhao H, Duarte J, Cascao R, et al (2010) Chronic arthritis leads to disturbances in the bone collagen network. Arthritis Res Ther 12: R9 doi: 10.1186/ar2908 20078856 10.1186/ar2908 PMC2875635

