# Supplementary figures and images for: Early arthritis induces disturbances at bone nanostructural level reflected in decreased tissue hardness in an animal model of arthritis
Source: PLoS One. 2018 Jan 9;13(1):e0190920. doi: 10.1371/journal.pone.0190920 (PMC5760022; doi:10.1371/journal.pone.0190920)

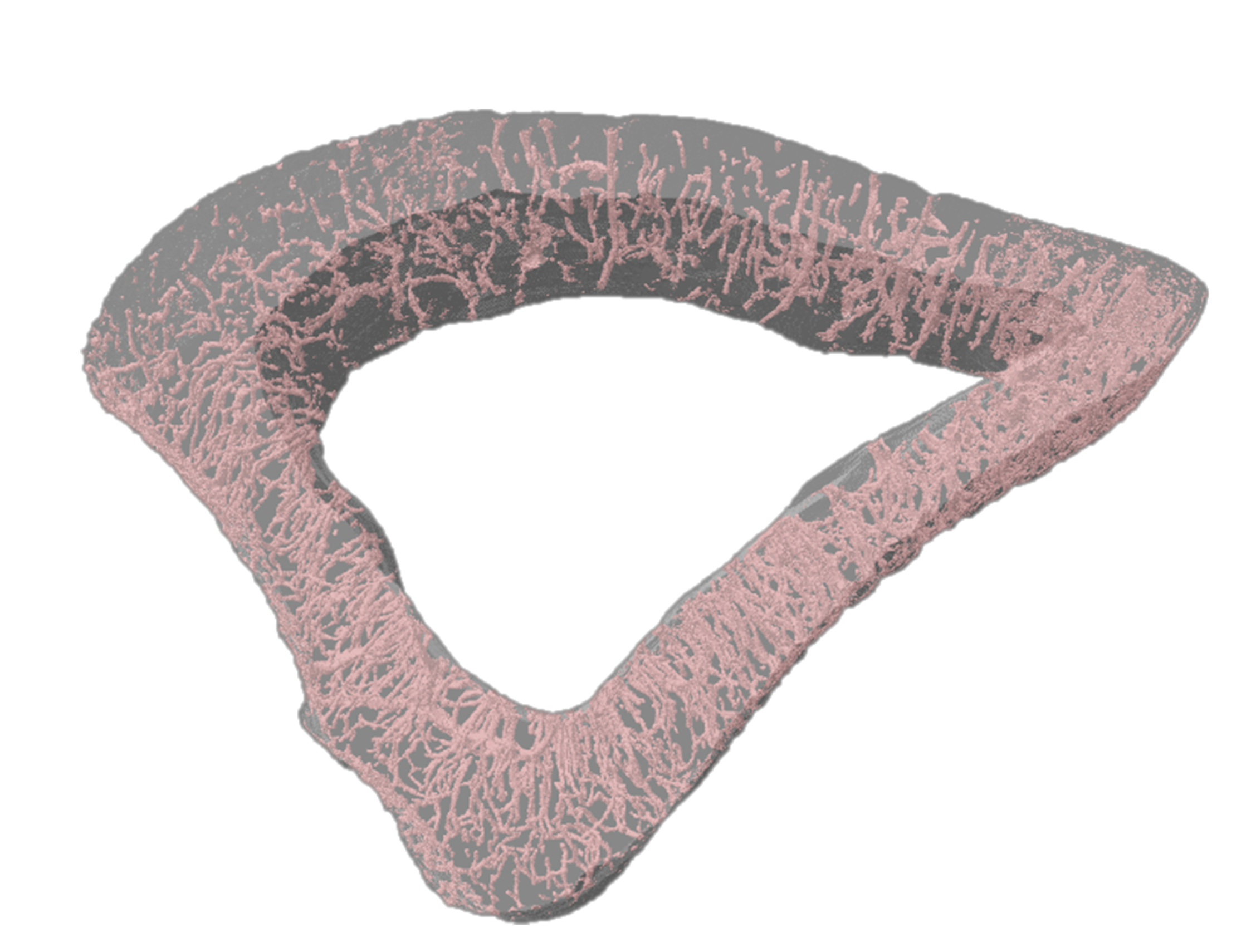

Supplement: S1 Fig — (TIF) [file pone.0190920.s001.tif]
